# ORIGINATION, DEVELOPMENT, AND VALIDATION OF THE SURGICAL PAUSE

**DOI:** 10.1093/geroni/igae098.1561

**Published:** 2024-12-31

**Authors:** Jason Johanning

**Affiliations:** University of Nebraska Medical Center, Omaha, Nebraska, United States

## Abstract

Frailty is a validated concept established in the geriatric literature identifying patients (regardless of age) at increased risk of dying within 6 months. Half of veterans older than 65 years seeking colorectal or cardiac surgery are frail or prefrail, markedly increasing the cost of care as well as the risks for postoperative mortality and complications. In this setting the Omaha VA implemented a quality improvement program adopting a 6 month mortality tool for surgical use given the controversy about how best to measure frailty. The results in Omaha were sufficiently impactful that work then focused on replicating and validating the use of the Risk Analysis Index in surgical populations. The survival advantage observed in Omaha was replicated in longitudinal data from 5 University of Pittsburgh Medical Center (UPMC) hospitals in an interrupted time series analysis demonstrating reduction in 1-year postoperative mortality from 20% to 16%, an absolute reduction of 4.2%. And exercise training for as little as 3 weeks before surgery achieved clinically significant improvements in endurance, gait speed and respiratory pressures—changes that are likely to speed recovery and improve outcomes. The model has now been replicated and deployed at over 50 VA Medical Centers as the Surgical Pause initiative as well as multiple academic and private settings. The Surgical Pause and RAI have recently been recognized by the John Eisenberg Patient Safety and Quality Awards as the National Level Innovation winner for 2023.

